# Artificial Intelligence for chemical risk assessment

**DOI:** 10.1016/j.comtox.2019.100114

**Published:** 2020-02

**Authors:** Clemens Wittwehr, Paul Blomstedt, John Paul Gosling, Tomi Peltola, Barbara Raffael, Andrea-Nicole Richarz, Marta Sienkiewicz, Paul Whaley, Andrew Worth, Maurice Whelan

**Affiliations:** a European Commission, Joint Research Centre (JRC), Ispra, Italy; b Aalto University, Espoo, Finland; c Leeds University, Leeds, UK; d Lancaster Environment Centre, University Lancaster, UK; e The Evidence-based Toxicology Collaboration at Johns Hopkins Bloomberg School of Public Health, Baltimore, MD, USA

## Abstract

As the basis for managing the risks of chemical exposure, the Chemical Risk Assessment (CRA) process can impact a substantial part of the economy, the health of hundreds of millions of people, and the condition of the environment. However, the number of properly assessed chemicals falls short of societal needs due to a lack of experts for evaluation, interference of third party interests, and the sheer volume of potentially relevant information on the chemicals from disparate sources.

In order to explore ways in which computational methods may help overcome this discrepancy between the number of chemical risk assessments required on the one hand and the number and adequateness of assessments actually being conducted on the other, the European Commission's Joint Research Centre organised a workshop on Artificial Intelligence for Chemical Risk Assessment (AI4CRA).

The workshop identified a number of areas where Artificial Intelligence could potentially increase the number and quality of regulatory risk management decisions based on CRA, involving process simulation, supporting evaluation, identifying problems, facilitating collaboration, finding experts, evidence gathering, systematic review, knowledge discovery, and building cognitive models. Although these are interconnected, they are organised and discussed under two main themes: scientific-technical process and social aspects and the decision making process.

## Highlights

• Artificial Intelligence (AI) has potential to improve chemical risk assessment (CRA) and associated regulatory decisions.
• AI could influence the scientific-technical evaluation process *and* the social aspects of the CRA decision making process.
• Systematic Reviews are among the CRA processes that will profit most from the application of AI techniques.
• Using AI as external objective monitor could make Weight of Evidence considerations less subjective and more trustable.
• AI could identify pitfalls not observable by individuals participating in the CRA process.

## 1 Introduction

**Fig. 1:**
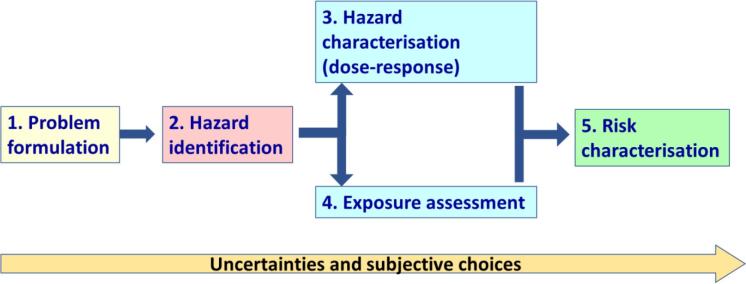
Steps in the Chemical Risk Assessment Process.

Chemical Risk Assessment (CRA) is a discipline at the science-policy interface that informs far reaching decisions about the placing of chemical compounds onto the market, thereby having a significant impact on a multi-billion industry, the health of hundreds of millions of people and the condition of the environment. The four elements of CRA, i.e. hazard identification, hazard characterisation, exposure assessment and ultimately risk characterisation (Fig. 1), depend on the integration of different types of information from different sources, and increasingly with an unmanageable volume of information, including regulatory dossiers, study reports and scientific literature. To add to the problem, there might be special interest influences, biased opinions, and political preferences (see the current glyphosate and acrylamide discussions). As a scientific body supporting the development of chemical risk assessment methodologies in the European Union, the European Commission's Joint Research Centre (JRC) has therefore launched and coordinated an initiative to explore the applicability of Artificial Intelligence (AI) to CRA as a means of supporting and improving regulatory decisions, and increasing public trust in the way the decisions are reached.

Similar efforts are being reported from the International Collaboration for the Automation of Systematic Reviews (ICASR) [3], [23], [24], which suggests a critical mass in interest in applying AI to assessments across environmental and biomedical disciplines. Establishing collaboration with ICASR to exploit a common focus will be a priority.

## 2 Workshop

A JRC-organised workshop, titled “AI4CRA – Artificial Intelligence for Chemical Risk Assessment”, was held in Ispra, Italy on 4–5 April 2018. The participants were scientists from both the AI and the CRA fields from Aalto University (Finland), Lancaster University, Leeds University (UK), the European Food Safety Authority (EFSA) and the JRC. The participants explored how, and to what extent, applying AI methods could support both the quantitative (the large number of insufficiently assessed chemicals and, the unmanageable extent of existing information sources) and the qualitative (speed, transparency, reproducibility, reliability, objectivity, credibility) aspects of CRA.

The following topics were identified as prime candidates to be examined more closely when further efforts are undertaken to make AI an enabling technology in CRA:

• identifying problems
• gathering evidence
• systematic review
• knowledge discovery
• supporting evaluation
• finding experts
• facilitating collaboration
• process simulation
• cognitive modelling

## 3 Further work

In the weeks following the workshop, the authors of this manuscript continued their discussion about the most promising areas that could be supported, facilitated or even made possible via the integration of AI. Based on the topics identified in the workshop, and following some regrouping, merging and additional input, two major overarching themes were identified:

• Scientific-technical evaluation process
• Social aspects and the decision making process

**Fig. 2:**
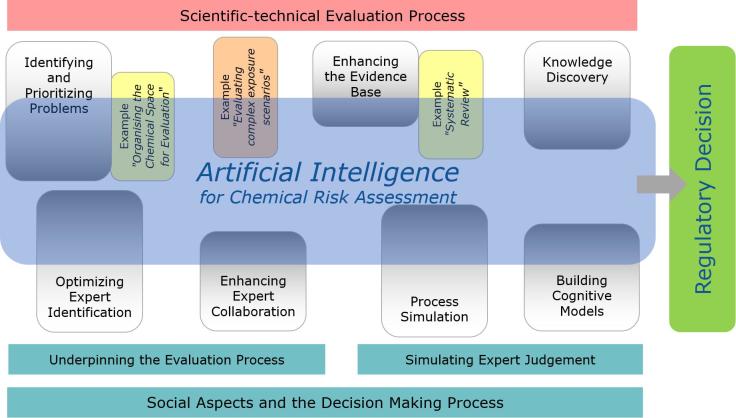
AI and Chemical Risk Assessment.

While the first theme touches upon more content-related issues like actually identifying problems to be addressed with CRA, gathering the scientific evidence via e.g. AI-supported systematic review and general knowledge discovery, the second focuses on the social interaction between actors and stakeholders by enhancing the human-led evaluation process and introducing the notion of simulating and exploring human expert judgement:

The level of AI-readiness of the individual topics (illustrated by their penetration of the AI area in Fig. 2) will also indicate the lower-hanging fruit and where quick wins can potentially be made. The ultimate goal of AI-powered Chemical Risk Assessment is of course the total overlap between the topics and AI.

## 4 Discussion - topics in chemical risk assessment that AI could support

### 4.1 Scientific-technical evaluation process

**Fig. 3:**
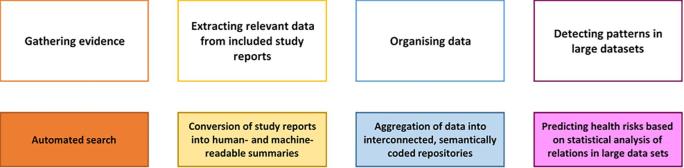
Possible roles of AI in CRA.

Fig. 3 illustrates several possible roles of AI in aggregating evidence and using the information to derive knowledge in the scientific-technical process. These are expanded upon below.

#### 4.1.1 Identifying and Prioritising Problems

Since a CRA can only assess what is targeted for evaluation, AI could be trained to find the gaps, based on the experience of previous CRAs, thereby identifying problems that are less obvious to experts.

For example, AI could help identifying the actual problems and questions to be asked in a CRA and screen and select/prioritise the chemicals to be assessed in the first place. AI could assist in identifying emerging risks, i.e. new adverse effects not typically covered by the standard regulatory endpoints.

Robust evidence surveillance methods are fundamental to evidence-informed risk management. Without robust evidence surveillance, it is not possible to know where there are significant evidence gaps and evidence gluts, and therefore not possible to efficiently prioritise where to commit limited research capacity to either synthesis existing evidence or to commission novel primary studies. The result of lack of robust evidence surveillance is inefficiency, as empty, inconclusive or irrelevant reviews might be undertaken, unnecessary primary studies might be commissioned, and necessary studies might remain unconducted.

In addition to analysing the evidence and supporting judgements, AI can support selecting and prioritising the chemicals for evaluation in the first place. A general challenge in CRA is the slow pace of evaluation of individual substances compared with the number of existing chemicals for which the safety of use, or respective risk management measures, have to be established. How to select the chemicals to evaluate first in the overall universe of chemicals? AI can help screen all available information or indications from different sources – which should comprise the whole universe of available information, including grey literature or social media – on properties and effects of chemicals, as well as the potential exposure to them, based on information on production, intended and actual uses. Starting with setting criteria for identifying the most critical chemicals, that is, posing the highest risk, or otherwise defined priority criteria, AI would learn and build indicators to flag priority chemicals for evaluation. Work of this nature has started on the high-throughput ToxCast data [17], [20].

A helpful concept in that context are evidence maps (also referred to as systematic maps or scoping reviews), which are gaining visibility as a useful systematic analysis product. In brief, evidence maps use systematic review methods to identify the amount and type of evidence available to address a particular topic [21], [33]. They may include critical appraisal of studies, but there is no attempt to synthesize the evidence to answer an assessment question. In this way, evidence maps also identify critical data gaps or reveal bodies of evidence where “traditional” evidence (experimental animal or epidemiological studies) is available for consideration, or when analysis tools for data poor chemicals would be more appropriate. Ideally, evidence is summarized in a structured format to promote dissemination in searchable databases and results are presented in a visual figure to keep the report concise. Evidence maps may be useful in the assessment update process to determine whether new evidence may result in a change to an existing health reference value. Increasingly, journals are providing author guidance on publication of evidence maps, including environmental journals [5], [8], [9]. With use of specialized systematic review software, in conjunction with AI tools for automating the mapping process, preparing evidence maps could be a rapid process (weeks, not months).

*Example 1: organising the chemical space for evaluation*

One approach to facilitate the evaluation of the high number of existing chemicals is the attempt to organise the chemical space and establish groups of similar chemicals to evaluate them together, in order to enable a faster and more efficient evaluation compared to single chemical evaluation [7], [13]. Computational approaches are already used for this purpose. An evaluation on whether AI can improve this process should be made.

#### 4.1.2 Enhancing the evidence base

The collation of relevant evidence and evaluation of its quality and suitability for use in the assessment process are crucial steps at the beginning of a CRA.

AI could be trained to crowd-source multi-disciplinary expertise, and add complementary and useful knowledge normally not considered in a specific context.

New streams of data from biomonitoring and epidemiological studies can contribute to the overall picture [22] but might be difficult to analyse “manually”. Here natural language processing could be employed to derive useable information from disparate types and sources of data (similar to the knowledge discovery employed in [18]. AI will be particularly useful for dealing with unstructured content.

In line with Wilson’s statement that “we are drowning in information, while starving for wisdom” [31], there are many different sources providing information for CRA and the amount of data is rapidly increasing, for example, using high throughput screening or omics technologies. These “raw data” have to be processed and interpreted to provide useful insights for CRA, which means that AI could filter out the relevant data and synthesis information to build knowledge.

Not only scientific literature, study reports and submitted dossiers should be considered for CRA, but other valuable “unofficial” sources of information should also be included and mined, such as “grey” literature and other information from the web and social media, e.g. twitter or blogs. These could contribute to identifying trends or emerging risks, by providing information for example on actual use (or misuse) of, and exposure to, chemicals.

AI could contribute to building good quality data for CRA. In addition to analysing evidence more efficiently, AI could identify data gaps and help to formulate the corresponding research questions. AI might contribute to setting up the experimental design in the most efficient and effective way to close the knowledge gaps.

The main obstacle to keeping on top of research is that scientific papers are being published at ever increasing rates. To keep abreast of all research which is relevant to a topic or policy area is impossible without some means of automating the aggregation and summarisation of the content. Without technology, research synthesis efforts remain limited and incomplete.

In the near term, AI machine-reading tools will enable the characteristics and results of hundreds of thousands of studies relevant to assessing chemical health risks, currently locked away in individual manuscripts, to be converted into composite, queryable databases. These databases can be set up with flagging systems for regulators, automatically identifying new evidence streams which should be analysed, and they should be queryable in terms of concepts essential to their decision-making process. This way, risk managers can be confident that they are acting on all relevant evidence, not just part of it, and their reasoning for focusing on one part of the evidence base rather than another can be made fully transparent.

*Example 2: systematic review*

In the near term, AI could dramatically increase the amount of conducted systematic reviews (SRs), which in the long run could lead to a culture of evidence-use based primarily on the systematic gathering and appraisal of evidence streams. This would reduce the risk of a 'single study' influence on policy decisions, and ease the process of obtaining scientific consensus. They are both vital for areas where facts are heavily contested and opinions highly polarised.

An SR aims to provide a complete summary of current evidence which is relevant to answering a research question. They are characterised by the effort made to minimise the risk of bias in the evidence review process, emphasising sensitive search strategies, comprehensive inclusion criteria, and transparent and reproducible approaches to critically appraising existing evidence and synthesising it into summary results (via qualitative and/or quantitative meta-analytical methods).

While the transparency and the minimised risk of bias of an SR has a strong appeal as a technique for developing the methodological quality of CRA, they are lengthy, labour-intensive projects to undertake, which require a great deal of specialised skill across multiple disciplines to complete successfully. This is proving a significant barrier to their uptake in regulatory contexts, where time-frames are often relatively short, and capacity often limited in comparison to the requirements of the methodology.

**Fig. 4:**
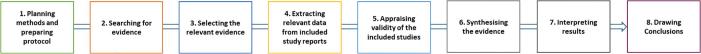
Steps of a Systematic Review.

AI stands to shift this balance in many ways, potentially at every stage of the review process. AI can facilitate each individual step (see Fig. 4) of an SR, lowering the entry barrier to the use of SR methods, therefore enabling their application in previously inaccessible contexts. The way individual studies are conducted and reported is also expected to change as AI becomes mainstreamed, with new reporting standards developed to facilitate automated search, selection, quality assessment and statistical synthesis.

*Example 3: evaluating complex exposure scenarios*

AI could help with complex evaluations, for example to conclude on possible effects of combined exposure to chemicals and to simulate exposure to chemicals for a multitude of different possible exposure scenarios and probabilities – across different sectors and pathways, such as via food, consumer goods, occupational and environmental exposure, and for different populations in terms of geography and susceptibility, and over different periods of time. Similarly, in the direct assessment of chemical mixtures, even in the ideal case that the effects were known for all individual chemical components, the sheer number of possible combinations of several compounds in many possible concentrations is too high as to allow a simulation of the mixtures and measurement of the effects of all these theoretically possible combinations in a laboratory [4]. AI could help with simulations of all possible mixture (compound and concentration) combinations and thus the prediction of their combined effects, or the determination of the worst case. AI might also contribute to predict interaction of chemicals.

#### 4.1.3 Knowledge discovery

As defined in the Commission Communication [11], knowledge “is acquired through analysis and aggregation of data and information, supported by expert opinion, skills and expertise”.

Information from distinct sources might give new insights when brought together. However, so far there might be no awareness about the existence of and/or connections between these different sources. AI could help to screen and systematically map the “universe of information”, extracting information also from non-easily processable sources, or hidden in scanned documents or graphs, and subsequently to combining them. Pattern recognition techniques can help to make connections and identify relationships, which would be too complex to be recognised by human screening and processing, and support discovering correlations. Furthermore, very different types of data/information have to be integrated to enable conclusions and decision making. AI could help by making sense of data and building knowledge. An example is the Adverse Outcome Pathway (AOP) approach for systematically describing mechanisms of chemical toxicity, and the possibility to find new AOPs or build interconnected AOP networks, based on many scattered pathways or fragments of pathways [32].

AI could be trained to identify other substances that could inform the CRA, if identification of similarity in chemical structures, biological behaviour or nature of the available data, would be made. Once enough data have accumulated in the chemical risks database, AI could be used to build multidimensional models of patterns latent within the data (such as trends in toxicity), and compare the “profiles” of different chemicals. By comparing such profiles, risk assessors will be able to base predictions of health risks such as carcinogenicity on the basis of all the available evidence, including hidden similarities.

The authors recognise multiple initiatives exist in this space, e.g. at ICASR, US EPA, NTP, who are working on operationalising crowd sourcing options with a focus on ensuring interoperability of tools, onboarding of AI, development of training data sets for model development, mapping of various vocabularies into an ontology framework that can be used to help with AOP development and other evidence synthesis activities, and incorporation of tenants of systematic review into the AI pipeline. As a result of the workshop, JRC is reaching out to various actors to encourage further collaboration and streamlining of efforts.

### 4.2 Social aspects and the decision making process

#### 4.2.1 Enhancing the evaluation process

After aggregating the evidence, the next step in a CRA is evaluation. The compiled lines of evidence are assessed, considering the quality of the data, their reliability and associated uncertainties, as well as the relevance for the problem formulated, in order to make an overall judgement. The assessment needs to integrate results from different types of approaches and should be performed in an unbiased way while weighing their respective contributions and handling possibly conflicting results. Evaluation is typically an expert judgement driven process, with expert knowledge and experience being required to make decisions regarding the available data and the conclusions based on them.

Different groups of experts are making CRAs in different legal frameworks based on the data made available at a certain point in time. In hindsight, it is often difficult to understand whether and what data was actually considered and how different data was weighed to lead to final conclusions and recommendations regarding the possible or restricted uses of a substance. The discrepancy between decisions taken by different expert groups can lead to mistrust in the resulting CRAs and expert recommendations. Generally, AI could play a role in carrying out the evaluation process in a more neutral, consistent and transparent way.

AI could learn how to perform a CRA which could then be peer-reviewed by real experts, and this would be the basis for an iterative learning process for both the machine and the experts.

##### 4.2.1.1 Optimizing expert identification

In terms of expert panel work, there are several ways in which AI could support the CRA process. For example, since the success of a group decision process depends on the dynamics of the group, as well as the complementary strengths and areas of expertise of its members and their knowledge of the topic, and on avoiding potential bias due to the experts’ background, finding an optimal mix of experts is a crucial task which AI could support. A selection based on limited or biased selection criteria, or only based on already established networks, can reduce the chance of finding the right balance of experience, competence and different views which are beneficial for a fruitful discussion. AI could be used to create a mapping system that takes into consideration not only the potential experts’ publications on the topic, but parameters that are normally not considered and difficult to retrieve manually, such as membership in other similar panels, presentations at conferences, presence in committees, etc. Combined with a broad and up-to-date search for the information to map the expertise, this process could reduce the risk of bias and broaden the pool of possibilities, not relying on personal experience or potentially biased judgement criteria of the selectors.

##### 4.2.1.2 Enhancing expert collaboration

Furthermore, AI could monitor or guide the process in an interactive Weight of Evidence (WoE) and thus help to rationalise the decisions made in the process. A possible direction is to use AI as an external objective observer. It could automatically create and maintain an organised, up-to-date record of topics discussed, and provide further context by linking to relevant information from available sources. This AI-driven external observation could take the form of a dynamic ‘mind map’, generated on the fly as the work of the group progresses, following and facilitating the process in a transparent way. AI would have the ability to cope with large amounts of data regarding individual chemicals, similar chemicals, chemicals in combination, experience made in earlier risk assessments, recognising differences between different evaluations of one chemical or of similar chemicals in different contexts.

AI as an instant feedback instrument could also point out all possible variables and their impact, the pros and cons of different options that the group may be faced with, as well as the consequences from the judgements made for the final outcome. This would include tools to explain consequences of decisions thereby informing the weighing of economic and health related impacts.

In terms of evaluating uncertainties in CRA, AI can take the probabilistic assessment to a next level by having uncertainties explicitly at the core of the risk assessment methodology.

Another direction is a more active involvement of AI in the group decision process, by directly interacting with the members of a group. The contribution of AI could even consist of being a full member of the expert team and contributing an additional expert opinion, drawn from the available data. Going beyond the role of making evaluation more efficient, AI could develop into an overarching universal expert entity in itself, by learning about human expert decisions based on the available evidence. AI would build a collective memory of and accumulate the experience from many experts and learnings from previous cases. Furthermore, AI could derive the implicit knowledge from experts in the first place. This could be valuable for industry and regulators because the potential loss from key experts missing risk assessments could be mitigated. The accumulated knowledge from all AI-assisted group decision processes could act as a shared memory or knowledge resource.

### 4.2.2 Simulating expert judgement

#### 4.2.2.1 Process simulation

Once the scope of a CRA is defined, the CRA process is based on the four steps of hazard identification, hazard characterisation, exposure assessment, and risk characterisation (Fig. 1). Since the risk assessor needs to make a number of methodological choices at each step, the CRA process can in principle follow a number of different pathways, leading to potentially different conclusions and risk management measures. In practice, the risk assessor reaches a conclusion based on just one of these pathways, without exploring or acknowledging the other possibilities. AI would provide a means of simulating all possible pathways, i.e. the “multiverse” of risk assessment outcomes for a given defined problem formulation [27], [29]. This would provide a means of characterising the uncertainties in the CRA process and provide the risk manager with transparent documentation on the range of possible CRA outcomes from the most to the least conservative assessment. These uncertainties cover not only issues related to experimental design and the inherent variability of experimental data, but also the impact of subjective choices, such as one data type over another, or one data analysis procedure over another. In the short term, simulating the CRA process would provide a means of judging the reliability of risk assessments already conducted, especially in the case of controversial chemicals of high societal concern, thereby informing actions to reduce specific uncertainties where these are deemed too large. In the long term, the ability to simulate the CRA process will largely obviate the need for the risk assessor, thereby freeing resources to place more emphasis on problem formulation, taking into account a wider range of chemical concerns than is addressed by the existing regulatory framework.

#### 4.2.2.2 Building cognitive models

Human processes, especially those related to decision making and government, are often assumed to be rational and logically structured. The calls for transparency of decision making are motivated by the need to scrutinise the process, i.e. to verify how much it follows this clear, predefined, logical pathway of reasoning. Certainly, this assumption of rationality is prevalent in strongly codified areas such as risk assessments, but also holds true in other complex co-decision processes where many sources of knowledge, actors and interests are involved.

However, the assumption that decision making is mostly rational and strongly relies on an objective analysis of facts is often discussed as simplistic [6], [19], [26]. Our rationality is more subjective and context-bound than can be assumed [10], [12], [16], and our values and emotions have an important influence on the perception of facts and willingness to act on them [1], [25], [28]. Moreover, our cognitive processes are influenced by cognitive biases, hence not all facts and all positions in the policy and decision making processes are equally considered (even if it seems so by involved actors), which affects the results [2], [14]. Therefore, decision making is not guaranteed to improve simply because more or better science is introduced to a process: the perception and interpretation of facts depends on many factors.

It is unlikely, however, that the decisions are made in a completely haphazard way. There must be underlying mechanisms of how they happen, with patterns of interacting factors contributing to particular results. Therefore, it can be beneficial to better understand our cognition and group dynamic in the context of chemical risk assessment, science advice and decision making. As risk assessment processes and precautionary principle are often contested, a constructive rethinking of them would benefit from a better understanding of the factors at play and their interconnections.

For complex processes, a single person, or even a team routinely involved in them, will have difficulty in systematically auto-analysing these patterns of human thinking, interacting, and decision making. This is where AI comes as a potential solution: it could help model cognitive and decision-making processes [15], [30]. Acting as a neutral observer, silently shadowing and recording the decision making steps, AI could model them and uncover the logic of their structure, not necessarily as rational as assumed. These objective models could identify pitfalls not observable by individuals participating in the process, spot the major bottlenecks and as such bring more awareness about the realities of the decision making, knowledge elicitation or risk assessment processes.

The objectives would slightly vary, depending on scenarios:

1. Where the rationality is strongly expected and clearly codified, AI observations could reveal the actual complications of these assumptions, pointing out areas worth improving in the process.
2. Where the process is already not fully clear (especially from an individual’s perspective in complex systems), they give a tool to understand what is going on. This could be highly interesting information e.g. for researchers interested in providing their input to policy effectively, as they often struggle with an overly simplified policy cycle model.

Models produced by AI could provide empirical evidence of the functioning of decision-making processes. Through such cognitive models, weaker and stronger points of complex processes can be identified and a more thorough reform can be foreseen. If focused specifically on scientific support, it could help map the best ways of achieving impact with evidence.

In practical terms, it would require a sufficient number of observable cases (e.g. with recording the meetings, mapping the information flows, related decision trees/patterns, etc.), which requires a lot of trust on the side of various institutions who manage or participate in these processes.

#### Step-by-step approach

Full exploitation of all benefits of AI for CRA cannot be a short term operation: Prior to use for decision-making, users will need to see evidence in the quality of AI for accurate collection of information reported in studies (both from journals and grey literature) as well as from data sources that may not be reported in single studies (high throughput screening results). Also, the development of AI-based evidence integration approaches will have to be done with awareness of structured frameworks being used in environmental health for weight of evidence (e.g., approaches developed by NTP, US EPA IRIS, GRADE).

## 5 Conclusions

The workshop participants concluded that AI can support and facilitate important processes and activities in the multifaceted CRA discipline. While the introduction of AI to support CRA will not be a trivial undertaking, the current problems that CRA is facing (inadequate resources, cases of information overload, public scepticism) will not be solved by conventional means. “More of the same” will not lead to better and reliable CRA, but AI offers the opportunity to support CRA in innovative ways. These include supporting the gathering and analysis of information as the basis for the CRA, facilitating different aspects of expert evaluation, including AI as an additional expert around the table, to simulating all possible assessment pathways and the range of possible outcomes, as well as creating new knowledge and cognitive models of decision-making.

AI assisted innovation in evidence gathering could therefore build an automated system of transforming data into knowledge, with much more efficiency. Freed up brain power could be used to identify important gaps in knowledge and frame the right questions. Altogether this leads to a more robust evidence base for policy making, and hopefully to a more transparent method in the decision making process.

Using AI is not only beneficial for humans, but in turn can help develop better AI. For example, it would allow developing better systems for human-AI collaboration and AI-based argumentation in the CRA process, building on the complementary strengths of human and machines. In particular, cognitive models not only help AI understand humans better (e.g., goals, preferences, decision making processes) but also help developing human-understandable and transparent algorithms that can explain reasons (including emotions) behind their actions.

It is expected that further exploration of the topics covered in this workshop could lead to executable plans for pilot projects, with a view to eventually increasing the efficiency and effectiveness of the CRA process. However, the only way to fully realise the applications of AI for CRA is global and discipline-crossing collaboration. The AI method development and deployment needs (including associated user dashboards) are too large and complex to be developed by any single entity. The development of AI should include a focus on interoperability of tools to best take advantage of the talent and resources available in both the environmental and biomedical sciences.

## Declaration of Competing Interest

The authors declare that they have no known competing financial interests or personal relationships that could have appeared to influence the work reported in this paper.
